# Detecting Concealed Information from Groups Using a Dynamic Questioning Approach: Simultaneous Skin Conductance Measurement and Immediate Feedback

**DOI:** 10.3389/fpsyg.2013.00068

**Published:** 2013-02-15

**Authors:** Ewout H. Meijer, Gary Bente, Gershon Ben-Shakhar, Andreas Schumacher

**Affiliations:** ^1^Faculty of Psychology and Neuroscience, Maastricht UniversityMaastricht, Netherlands; ^2^Department of Psychology, The Hebrew University of JerusalemJerusalem, Israel; ^3^Department of Social Psychology and Media Psychology, University of CologneCologne, Germany

**Keywords:** lie detection, concealed information test, guilty knowledge test, searching concealed information test

## Abstract

Lie detection procedures typically aim at determining the guilt or innocence of a single suspect. The Concealed Information Test (CIT), for example, has been shown to be highly successful in detecting the presence or absence of crime-related information in a suspect’s memory. Many of today’s security threats, however, do not come from individuals, but from organized groups such as criminal organizations or terrorist networks. In this study, we tested whether a plan of an upcoming mock terrorist attack could be extracted from a group of suspects using a dynamic questioning approach. One-hundred participants were tested in 20 groups of 5. Each group was asked to plan a mock terrorist attack based on a list of potential countries, cities, and streets. Next, three questions referring to the country, city, and street were presented, each with five options. Skin conductance in all five members of the group was measured simultaneously during this presentation. The dynamic questioning approach entailed direct analysis of the data, and if the average skin conductance of the group to a certain option exceeded a threshold, this option was followed up, e.g., if the reaction to the option “Italy” exceeded the threshold, this was followed up by presenting five cities in Italy. Results showed that in 19 of the 20 groups the country was correctly detected using this procedure. In 13 of these remaining 19 groups the city was correctly detected. In 7 of these 13, the street was also correctly detected. The question about the country resulted in no false positives (out of 20), the question about the city resulted in two false positives (out of 19), while the question about the streets resulted in two false positives (out of 13). Furthermore, the two false positives at the city level also yielded a false positive at the street level. Even though effect sizes were only moderate, these results indicate that our dynamic questioning approach can help to unveil plans about a mock terrorist attack.

## Introduction

The Concealed Information Test (CIT; Lykken, 1959; Verschuere et al., 2011) uses physiological responding to determine the presence or absence of crime-related information in a suspect’s memory. In a typical CIT, questions concern crime details known only to the perpetrator and the investigative authorities, but not to an innocent suspect. With each question, several answer options are presented serially, while peripheral autonomic nervous system activity is recorded. Answer options include the correct, but also several plausible but incorrect ones (e.g., “Was the victim dumped … (a) on a construction site, (b) in a pond, (c) on a beach, (d) in a dumpster, (e) in the trunk of a car”). For an innocent suspect, all options are equally plausible and will therefore elicit similar physiological responses. For a guilty suspect, the correct option is salient and significant, and will therefore elicit an enhanced orienting response (Verschuere et al., 2004). Such an orienting response is reflected by several psychophysiological responses, such as an increased skin conductance response (SCR; Lynn, 1966). Thus, a consistent pattern of stronger responding to the correct options indicates knowledge of intimate crime details, from which guilt can be inferred.

Historically, the CIT has been used to infer guilt or innocence using information known to the investigative authorities. However, the CIT can also be employed when the correct option is not known, and the purpose of the investigation is to detect which of several options is the correct one. In this case, a series of options is presented to the suspect, and the option that evokes the largest physiological response warrants further investigation. This approach is often referred to as the Searching-CIT (S-CIT; Osugi, 2011) and can be used to discover, for example, the location of the body of a murder victim when the perpetrator is known (Nakayama, 2002). Applying the S-CIT to a terrorism scenario, Meixner and Rosenfeld (2011) asked 12 participants to choose a type of bomb, a location, and a date for a mock terrorist attack from a list, resulting in 36 to be detected details. Using a CIT based on the P300 component of the event related potential, they were able to correctly identify 21 out of these 36 details, with no false positives.

Meijer et al. (2010) applied a variant of the S-CIT to a group of mock terrorism suspects. The idea behind this study was that the CIT and S-CIT are typically used to render a decision at the individual level. Yet many of today’s security threats come from terrorist networks and organized crime. In these cases there may often be a group of people suspected of either planning or committing a crime. In Meijer et al. (2010), 12 participants were instructed to pretend they were members of a terrorist organization. They received information about the target, location, and date of an upcoming terrorist attack, and were then subjected to the CIT. An analysis at the group level showed that the correct option elicited a significantly larger average SCR, and as such information about an upcoming mock terrorist attack could be extracted from the group. Using a similar group approach but with a standard CIT, Bradley and Barefoot (2010), tested whether they could correctly identify exposure to one of three mock village scenarios. Groups of participants viewed tea making, bomb-making, or no activity, while building a card house. The CIT results showed that on the basis of group average SCRs, 80% of the bomb-making groups, and 75% of the tea making groups were correctly identified.

While Meijer et al. (2010) showed that the CIT can be used to elicit sensitive information from groups, their approach may be of limited applicability because the CIT format requires a limited number of plausible answer options. In some cases the number of available options may be naturally limited; while in others, the available options could be reduced by police work. Yet, the potential for real life application of the group variant of the S-CIT would be increased considerably if the content of test questions administered to suspects could be made contingent on their physiological responding to previous questions. For example, if the location of an upcoming attack is of interest, the first question could entail different countries, the next question could entail regions, then cities, etc. However, using series of questions requires an immediate feedback about which option evoked the largest mean physiological response. In the current experiment, we tested whether such an approach could be used to identify details of a mock terrorist attack. To enable immediate feedback, we performed an experiment in which we simultaneously measured skin conductance of groups of participants. These group data were analyzed immediately after each question, and the next CIT question presented was selected based on the responses to the previous question.

## Materials and Methods

### Participants

Participants were 105 students of the University of Cologne, who received 10€ for their participation. Participants were tested in groups of five. Data of one group was discarded due to technical failure. Thus, the remaining sample consisted of 100 participants (28 men) with a mean age of 23.7 years (SD = 3.66).

All participants received written information about the procedure of the experiment before coming to the lab and read and signed a letter of informed consent before participating. The experiment was approved by the ethical committee of the Faculty of Psychology and Neurosciences of Maastricht University.

### Procedure

Once all five participants of a group arrived in a room located next to the laboratory, they were instructed by the experimenter to treat the experiment as a role playing game and imagine being members of a terrorist network whose job is to select a location for an attack. No reference to the type of attack was made. The group was informed that once they had selected their location, they would be subjected to a lie detection test, and their task was to try to conceal the information from the experimenter. The experimenter stressed that it was crucial to the study that everybody remembered their choice, and that they would be given a memory check after the test. Next, the experimenter instructed the group on how to select a location, and left the room. The group was given 10–15 min to make their selection.

The location of the attack consisted of a country, a city within this country, and a street within this city. First, the group had to open a sealed envelope labeled “Countries.” This envelope contained a list with five European countries. Together, they had to decide on a country for their attack. Next, they opened a second envelope which contained five separate envelopes, one for each country. They opened only the envelope for the country they had chosen, and this envelope contained a list of five cities within that country. They chose one of these cities, and proceeded with opening the last envelope labeled with the city of their choice. This last envelope contained a list five streets in the chosen city, from which they selected one. This procedure was used to ensure participants were not exposed to the cities and streets that were not part of their chosen location^1^. Once the group had selected their location, they listed it on a form signed by all members. This form served as the ground truth criterion. One member of the group held on to this form, and gave it to the experimenter at the end of the experiment. Thus, the experimenter was unaware of the details the group had chosen.

Once the group had completed the steps described above, they came to the testing room where the experimenter was waiting. Participants were seated in five cinema chairs facing a wall, and separated by room dividers so they could not see each other. Sensors measuring skin conductance were attached, and the S-CIT was performed. During the S-CIT, the experimenter was seated behind the cinema chairs. Upon completion of the CIT, the participants filled out a free recall memory check, and were thanked and paid for their participation.

### Searching-concealed information test

The S-CIT consisted of one example question and three test questions. The example question dealt with the day of the week (Today is … *Monday* … *Tuesday* … *Wednesday* … *Thursday* … *Friday* …) and served to familiarize the participants with the procedure. Test questions referred to the country (“With this question we will determine in which country the attack will take place. Is it …?”), the city (“With this question we will determine in which city the attack will take place. Is it …?”), and the street (“With this question we will determine at which street the attack will take place. Is it …?”). Each question was presented for 10 s and followed by six options, each presented for 7 s. A random Inter Stimulus Interval ranging between 16 and 24 s was used. The first option presented within each question served as a buffer, and was excluded from all analyses. The following five options were presented in a random order. These five options were identical to the five options the group could choose from during the planning phase. Examples of options are France, The Netherlands, Belgium, Italy, and England for the countries, Reims, Bordeaux, Lille, Marseille, and Toulouse for the cities and Rue de Vesles, Rue Buirette, Rue de L’etape, Rue Carnot, and Rue des Murs for the streets. Obvious options such as capital cities and well known streets were avoided. Each question was repeated a number of times, depending on the outcome (see below). All stimuli were presented in a bimodal fashion; auditory via headphones; and visual text projected on the wall using a beamer. Each participant received a slider box, and was instructed to push this slider down with their right hand representing a “no” answer. This was done to encourage participants to focus their attention to the test. No data were, however, recorded from these slider boxes.

### Skin conductance measurement, response scoring, and analysis

Skin conductance was measured using dry electrodes with 1V DC system (Wild devine IOM), and sampled at 31 Hz. Sensors were placed on the tip of the index finger and the ring finger of the left hand of each participant. SCR’s were defined as the maximum positive deflection in the 1–7 s window after stimulus onset. To eliminate individual differences in responsivity, the raw SCRs were transformed to a within-participants standard scores (Ben-Shakhar, 1985). Specifically, the SCR to each option was standardized relative to the mean and standard deviation of the SCRs across all five options within each question. Next, the *z*-scores for each option were averaged across the five participants, yielding a single *z*-score for each option. These *z*-scores were then averaged across repetitions.

The analysis described above was performed after each question, and the outcome was used by the experimenter to determine the next question to be presented. The following *a priori* rule was used to determine the choice of the follow-up questions. Each question was repeated twice. If after these two repetitions the average *z*-score of one option exceeded 0.4, this option determined the following question. If more than one option exceeded the 0.4 threshold, the option yielding the largest *z*-score was followed up. If no option exceeded the threshold, the question was repeated for a third time, and the option exceeding an average of 0.4 was followed up. If still no option exceeded the threshold, the test was stopped, and the verdict deemed “no decision.”

## Results

**Figure 1:**
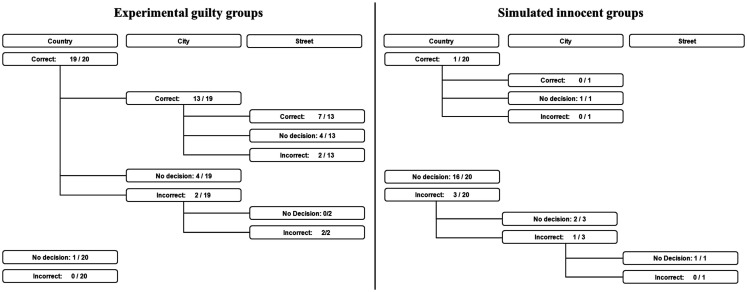
**Number of correct, incorrect and no decision verdicts for the three questions for the experimental participants (left panel) and the simulated innocent participants (right panel)**.

Correct recall on the memory check after the test was 100%. Average number of repetitions for question 1 was 2.05, for question 2 2.32, and for question 3 2.47. The results of the experimental groups are displayed on the left panel of Figure 1. The country was correctly identified in 19 of the 20 groups and in the remaining group no decision was made. The results of the second stage revealed that among the 19 groups for which the country was correctly detected, the city was correctly identified in 13. In four groups, no decision was made, while in two an incorrect option exceeded the threshold. Among these 13 groups, the street was correctly identified in seven, while in four groups no decision was made, and in two an incorrect street name exceeded the threshold. In the two groups where an incorrect city was identified and consequently followed up, an incorrect street exceeded the threshold. When averaging over repetitions, the question containing the correct alternative was presented to a group in 52 cases (20 for the country, 19 for the city, and 13 for the street). In 39 of these cases (75%) the correct option was identified. In 9 cases (17.3%) a “no decision” verdict was rendered, and in 4 cases (7.7%) an incorrect option was identified. In the 2 cases where a question without the correct option was presented an incorrect option was identified. For 7 out of the 20 groups (35%), the correct location (country, city, and street) was successfully identified, for 9 groups (45%) a “no decision” verdict was rendered, while in 4 (20%) an incorrect location was identified.

To compare these results with outcomes that would be expected under a condition of chance level performance, we applied the simulation procedure outlined by Meijer et al. (2007). Adopting this procedure to the present data, we randomly drew five values from a standard normal distribution, representing one participant’s responses to the five options of one question. These values were analyzed using the same steps used for the analysis of the experimental participants’ data, i.e., each of these five values was standardized relative to the mean and standard deviation of all five values. This was repeated five times representing a group of five participants. Next, the standardized values were averaged across these five “participants,” yielding a single *z*-value for each “option.” This entire procedure was repeated representing repetitions, and the *z*-values were averaged across two “repetitions” if one of the values was greater than 0.4, and averaged over three “repetitions” if no value exceeded 0.4.

Repeating this simulation for 10,000 groups of five participants yielded a “no decision” verdict in 78.8% of all simulations, while in the remaining 21.2%, one option was identified. Among these 21.2% of the simulations, for which an option was identified, it was the correct one in 4.2%, and the incorrect one in 17%. These percentages are displayed on the right panel of Figure 1 (as applied to 20 groups, rounded off to the nearest integer) such that they can be compared with the results obtained for the experimental groups. For example, while in 35% of the experimental groups the precise location (country, city, and street) was correctly identified such perfect identification was not obtained in any of the simulated groups.

To compare these results with those reported in other studies we computed the effect size based on individual data, using the ground truth criterion. This was done by simulating data to represent an innocent group that was matched to the experimental data, using the procedure outlined by Meijer et al. (2007). For example, the experimental data for question 1 (country) consisted of 95 participants (individual data for one group was not recoverable) of whom 90 were presented with two repetitions and 5 were presented with three repetitions. An innocent group consisting of the same number of participants and the same number of repetitions per participant was simulated. For each question only the groups for which the correct option was actually presented were included. Cohen’s *d* was calculated by subtracting the mean *z*-value of a randomly chosen option for the simulated data from the mean *z*-score to the correct option for the experimental participants and dividing this difference by the pooled standard deviation. Question 1 (Country; 95 participants) yielded an effect size of 1.12. Question 2 (City; 95 participants) yielded an effect size of 0.53. Question 3 (Street; 65 participants) also yielded an effect size of 0.53.

Finally, effect sizes (Cohen’s *d*) comparing the group averaged *z*-values were computed, including only those groups for whom the question with the correct option was presented. Effect sizes were 2.70 for question 1 (Country; 20 groups), 1.67 for question 2 (City; 19 groups), and 1.66 for questions 3 (Street, 13 groups). To check for the effect of habituation, we also compared the group averaged *z*-values of the first and the second repetition within each question using paired *t*-tests. Only for question 2 was there a significant decrease in differential responding between the two repetitions [*t*(18) = 2.27, *p* = 0.04]. The decrease in differential responding between the repetitions in question 1 and 3 were not significant [*t*(19) = 1.50, *p* = 0.15 and *t*(12) = 1.77, *p* = 0.10, respectively]. Effect sizes of the group averaged *z*-values based on only the first repetition decreased to 2.15 for question 1, 1.36 for question 2, and 1.29 for question 3.

## Discussion

The goal of this experiment was to examine the possibility of applying a variant of the S-CIT to detect concealed information from groups of suspects using a sequence of questions, such that the content of a question is contingent on the physiological responding to the previous question. To enable immediate feedback, we collected skin conductance data simultaneously from multiple participants and analyzed the responses immediately following each question. Results showed that the precise location of a mock terror attack planned by the participants was correctly detected in 35% of the groups, while in 20% an incorrect location was identified. The remaining groups (45%) rendered a “no decision” verdict.

Although the procedure performed above chance level, it led to a relatively high number of incorrect identifications. Two considerations are important here. First, it is important to realize that in the four groups where an incorrect location was identified, the information was still partially correct. In two groups the Country was correctly identified, while in the other two both the Country and the City were correctly identified. As such, the test did yield some information gain. Second, in contrast to criminal investigations where the CIT outcome typically addresses the guilt or innocence of a suspect, in the current application of the S-CIT, the costs of missing information about a planned terror attack outweigh the costs of incorrectly identifying a location. Even though due to the design of the current study, an incorrect identification also means missing the correct option, this may not be the case in a real life application, as the correct option may simply not be included. This justifies the use of a non-conservative cut-off point, yielding a relatively large false positive rate as done in this study. Yet, it is important to realize that other applications may warrant a different cut-off point than the one used in this study.

The relatively high number of false identifications and no decisions verdicts is not surprising given that the effect sizes at the individual level were only moderate. A number of explanations may account for this. First, the sensors used were dry electrodes, which may be less sensitive to changes in skin conductance. Secondly, the stimuli may have possessed relatively little signal value. Contrary to mock crime studies, where participants actually perform an act, in the current study participants were required just to pick options from a list. Needless to say, they were aware of the fact that they would not actually act out the scenario. In this sense the paradigm used here resembles the card test or the code words paradigms, which have been shown by Ben-Shakhar and Elaad (2003) to yield effect sizes (1.35 and 1.16, respectively) of similar magnitudes to those obtained in this study for the first question (1.12).

The first question yielded a higher effect size than the second and third question. Several explanations may be offered for this finding. First, due to habituation differential responding to correct and incorrect options may have decreased over time. Yet, the analysis of the repetitions within each question did not yield strong support for this. Although there was a significant reduction in differential responding for question 2, the effect sizes of all three questions decreased when using only the first repetition, due to the increased standard deviation in the simulated innocent group. An additional explanation for the difference between the questions may be found in the work of Bradley and Janisse (1979). These authors used a standard CIT, with pupillary response as the dependent measure. By giving the participants fake feedback about the test’s performance during previous trials, participants were led to believe that the test was either perfectly effective (100% accurate), somewhat effective (33 or 67% accurate), or perfectly ineffective (0% accuracy). Results showed that participants who were led to believe that the test was somewhat effective were easier to detect than those who were led to believe that the test was perfectly effective or perfectly ineffective. In the current experiment, in 19 out of the 20 groups, the cities presented with the second question were in the correct country. This may have served as feedback that the test was accurate to 95% of the participants, which, in line with the findings of Bradley and Janisse (1979) would explain the lower accuracy of the second and third question. Finally, the advantage of the first question over the subsequent questions may be explained in terms of differences in stimulus significance. Names of countries, may have been simply more salient and significant for the participants than names of cities and the streets.

Several limitations of this study deserve some attention. First, because the results were analyzed at the group level, all options need to be identical for all participants. As a consequence we did not check whether some of the items were personally relevant to some of the participants. But this will also characterize realistic situations. Secondly, as the experimenter was blind to what happened during the planning phase, we did not collect any data on social group interaction such as communication and compliance to the final decision. Future studies may incorporate such information, and, for example, test its influence on test efficiency. Finally, in the current experiment we used only guilty participants. One may argue that this does not represent realistic situations, where typically some suspects may be innocent and thus not possess any critical information. Thus, in reality the group tested may consist of both informed and uninformed suspects. Recently, Breska et al. (2012) tested the efficiency of two classes of algorithms for analyzing S-CIT data designed to detect critical information and differentiate between guilty and innocent examinees. The first class relied on a simple averaging procedure, while the second class relied on a PCA approach. They applied these algorithms on three data-sets of previous studies that used the standard CIT and demonstrated that in most cases the detection efficiency of both classes of algorithms was similar to that of the standard CIT. Moreover, the algorithms were relatively robust to the introduction of unknowledgeable participants in the sample. Such an analysis could also be applied with our dynamic questioning approach if only some participants possess the relevant knowledge.

The aim of our dynamic questioning approach was to increase the potential for real life application of the group variant of the S-CIT. Yet, due to the nature of the CIT format, even with this dynamic questioning approach the number of potential options needs still be limited somehow. Practically, this can be done by using intelligence gathered by investigative authorities. So it is important to note that even the dynamic questioning approach cannot be applied without at least some prior intelligence.

In sum, this study was a first attempt to use a dynamic questioning approach and despite the modest effect sizes obtained, and the finding that in only 35% of the groups tested the entire plan was correctly identified, we did demonstrate that this usage of the S-CIT can perform above chance level and yield important information gain. Moreover, even with the modest effect size of 1.12, the question referring to the Country of the attack yielded an impressive detection rate of 19 out 20 correct identifications. Although we can only speculate about the magnitude of the effect size to be expected in a field application, the bulk of available research indicates it will most likely be higher than the 0.53 obtained for the questions referring to the City and the Street (Ben-Shakhar and Elaad, 2003). We therefore believe that this approach deserves further research, for example with the use of multiple physiological and behavioral measures which can enhance detection efficiency.

## Conflict of Interest Statement

The authors declare that the research was conducted in the absence of any commercial or financial relationships that could be construed as a potential conflict of interest.

## Footnotes

^1^This also means participants were exposed to all the correct and incorrect options of their plan. Research by Verschuere and Crombez (2008), however, showed that such previewing did not affect detection efficiency.
